# A novel prognostic index for sporadic Burkitt lymphoma in adult patients: a real-word multicenter study

**DOI:** 10.1186/s12885-021-09144-1

**Published:** 2022-01-07

**Authors:** Mei-ting Chen, Fei Pan, Yung-chang Chen, Wei Zhang, Hui-juan Lv, Zhao Wang, Huang-ming Hong, Xiao-jie Fang, Ya-wen Wang, Tao Pan, Li-qun Zou, Hong-qiang Guo, Ke Xie, Li-min Chen, Xiao-qian Li, Yu-yi Yao, Ze-geng Chen, Hua-wei Weng, Xu-dong Li, Yuan-yuan Shen, Hui Zhou, Hong-wei Xue, Hui-lai Zhang, He Huang, Tong-yu Lin

**Affiliations:** 1 Department of Medical Oncology, Sun Yat-sen University Cancer Center, State Key Laboratory of Oncology in South China, Collaborative Innovation Center for Cancer Medicine, No. 651, Dongfeng East Road, Yuexiu District, Guangzhou, 510060 China; 2 Sichuan Cancer Hospital & Institue, Sichuan Cancer Center, School of Medicine, University of Electronic Science and Technology of China, Chengdu, 610041 China; 3 Departments of Lymphoma, Tianjin Medical University Cancer Institute and Hospital, National Clinical Research Center of Cancer, Key Laboratory of Cancer Prevention and Therapy, Tianjin’s Clinical Research Center for Cancer, the Sino-US Center for Lymphoma and Leukemia Research, Tianjin, China; 4 Department of Oncology, Affiliated Hospital of Qingdao University, Qingdao, Shandong 266003 P.R. China; 5 Affiliated Cancer Hospital of Xiangya Medical School, Central South University / Hunan Cancer Hospital, Changsha, 410013 China; 6 Department of Medical Oncology, Cancer Center, West China Hospital of Sichuan University, Chengdu, 610041 China; 7 The Affiliated Cancer Hospital of Zhengzhou University, Henan Cancer Hospital, Zhengzhou, China; 8 Department of Oncology, Sichuan Provincial People’s Hospital, Chengdu, P.R. China

**Keywords:** Sporadic Burkitt lymphoma, Adult, Pretreatment inflammatory biomarkers, Prognostic index, Real-word multicenter study

## Abstract

**Background:**

Adult sporadic Burkitt lymphoma (BL) is a rare but highly aggressive subtype of lymphoma which lacks its own unique prognostic model. Systemic inflammatory biomarkers have been confirmed as prognostic markers in several types of malignancy. Our objective was to explore the predictive value of pretreatment inflammatory biomarkers and establish a novel, clinically applicable prognostic index for adult patients with sporadic BL.

**Methods:**

We surveyed retrospectively 336 adult patients with newly diagnosed sporadic BL at 8 Chinese medical centers and divided into training cohort (*n* = 229) and validation cohort (*n* = 107). The pretreatment inflammatory biomarkers were calculated for optimal cut-off value. The association between serum biomarkers and overall survival (OS) was analyzed by Kaplan–Meier curves and Cox proportional models. The risk stratification was defined based on normal LDH level, Ann Arbor stage of I and completely resected abdominal lesion or single extra-abdominal mass < 10 cm.

**Results and conclusions:**

Univariate and multivariate analyses revealed that platelets< 254 × 10^9^/L, albumin< 40 g/L, lactate dehydrogenase≥334 U/L independently predicted unfavorable OS. We used these data as the basis for the prognostic index, in which patients were stratified into Group 1 (no or one risk factor), Group 2 (two risk factors), or Group 3 (three risk factors), which were associated with 5-year OS rates of 88.1, 72.4, and 45%, respectively. In the subgroup analysis for high-risk patients, our prognostic model results showed that high-risk patients with no more than one adverse factor presented a 5-year survival rate of 85.9%, but patients with three adverse factors had a 5-year survival rate of 43.0%. Harrell’s concordance index (C-index) of the risk group score was 0.768. Therefore, the new prognostic model could be used to develop risk-adapted treatment approaches for adult sporadic BL.

**Supplementary Information:**

The online version contains supplementary material available at 10.1186/s12885-021-09144-1.

## Introduction

Burkitt lymphoma (BL) is a rare but highly aggressive subtype of non-Hodgkin lymphoma (NHL) with the genetic hallmark of MYC gene translocation, including three different variants, namely, endemic, sporadic and immunodeficiency associated [1–3]. Endemic BL is associated with malaria and Epstein-Barr virus (EBV) [4]. The immunodeficiency-related variant has close relationship with human immunodeficiency virus (HIV) [5]. Sporadic BL is typically seen in young patients, but accounts for approximately 1% of adult NHLs [6].

With intense chemotherapy treatment, disease prognosis is excellent in children but poor in adults [7]. Acute treatment-related toxicity, such as severe myelosuppression, is an important factor affecting treatment outcomes in adults [8]. Mark Roschewski et al. demonstrated that dose-adjusted etoposide, doxorubicin, cyclophosphamide, vincristine, prednisone, and rituximab (DA-EPOCH-R) could avoid the need for high-dose intensive chemotherapy in adults with BL [9]. The International Prognostic Index (IPI) is frequently used for prognostication in many aggressive lymphomas, but is not commonly used in BL [10]. According to the Murphy staging system, childhood BL can be staged and classified as low or high risk based on the number of involved sites, presence of bulky disease, and lactate dehydrogenase (LDH) [11]. However, there is no standardized prognostic model for adult BL. Recently, in a multicenter real-world study in Western countries presented that ages≥40, Eastern Cooperative Oncology Group (ECOG) performance status (PS) ≥ 2, LDH 3 times more than the upper normal limit and central nervous system (CNS) involvement predicted inferior survival and constructed a BL-IPI in adult BL [12, 13]. But the prognostic model for adult patients with sporadic BL in Asian patient is still under establishment.

There is evidence that the inflammatory response plays a key role in different stages of tumor development, including initiation, promotion, invasion and metastasis [14]. However, few studies have focused on inflammatory biomarkers in BL. Wang et al. showed that low lymphocyte to monocyte ratio (LMR) was an independently adverse prognostic factor in 62 adult patients with sporadic BL [15]. Thus, it is important to identify inflammatory biomarkers to estimate prognosis more precisely.

In the current study, we performed a multicenter retrospective analysis to identify inflammatory biomarkers to predict survival in 336 adult patients with sporadic BL. A novel prognostic model was constructed to provide additional information for physicians making decisions regarding treatment options.

## Materials and methods

### Eligibility criteria and study population

We retrospectively surveyed 229 adult patients with newly diagnosed sporadic BL who were treated from August 2008 to September 2019 at Sun Yat-sen University Cancer Center, Sichuan Cancer Hospital, West China Hospital and Sichuan Provincial People’s Hospital in the training cohort. Eligible patients had histologically confirmed sporadic BL according to the 2008 World Health Organization (WHO) criteria, and complete clinical, laboratory, and follow-up data. All patients were confirmed diagnosed of BL by experienced pathologists. In order to avoid inclusion of high levels of B-cell lymphoma, all cases had been detected by fluorescence in situ hybridization for c-Myc, Bcl-2, and Bcl-6. Evaluation included standard laboratory tests, 18F-labeled fluorodeoxyglucose positron emission tomography (18F-FDG PET)-computed tomography (CT) scans of the whole body, and bone marrow aspiration and biopsy. Their medical records were analyzed, a prognostic model for sporadic BL was constructed, and we validated the results in a validation cohort. The validation cohort was retrospectively recruited from Tianjin Medical University Cancer Institute, Affiliated Hospital of Qingdao University, Henan Cancer Hospital and Hunan Cancer Hospital in China. The study protocol was approved by the ethical committee of Sun Yat-sen University Cancer Center (approval number SZR2019–016). Informed consent was not required because this study was a retrospective report of cases, which is a retrospective analysis of clinical data with no relevant to human biological ethic problems. The need of informed consent was waived by the ethical committee of the Sun yat-sen University Cancer Center. The study protocol was approved by the Institutional Review Board of the Sun yat-sen University Cancer Center and the study was performed in accordance with the principles of the Declaration of Helsinki. All methods were performed in accordance with the relevant guidelines and regulations.

### Data collection

The following baseline clinical information was extracted from electronic medical records (EMRs). Patient characteristics included age, sex, ECOG PS, Ann Arbor stage, B symptoms, pathological diagnosis, risk stratification, EBV-encoded DNAs, bone marrow status, and CT or magnetic resonance (MR) images of the neck, nasopharynx, abdomen, chest and pelvis or PET/CT of the entire body. Patients were assigned risk group according to the definition by Mead et al. [16]. Patients with low-risk had all of the following features: (1) normal LDH level, (2) Ann Arbor stage of I and completely resected abdominal lesion or single extra-abdominal mass < 10 cm; all other patients were considered high-risk.

The platelet, lymphocyte, monocyte, and neutrophil counts, LDH, C-reactive protein (CRP) and albumin (ALB) were collected from the last blood test before treatment. We calculated the neutrophil-to-lymphocyte ratio (NLR), derived neutrophil-to-lymphocyte ratio (d-NLR) and LMR. OS rates were selected as primary endpoints. OS was defined as the time between the date of diagnosis and the date of death or last follow-up.

### Statistical analysis

Categorical characteristics were compared using a chi-square test. PLT, LMR, NLR, d-NLR and LDH were calculated with receiver operating curves (ROC) to determine the optimal cutoff values and then dichotomized into 2 categories: less than, and greater than or equal to the cutoff values. The normal upper and lower limit for PLT was 100 to 350 × 10^9^/L and the normal lower and upper limit for LDH was 120 to 250 U/L. The optimal cut-off value of CRP and ALB are their clinical standard values. We applied the Kaplan-Meier method to perform survival analysis. Univariate Cox regression analyses and multivariate proportional hazards regression models were carried out to identify independent prognostic factors. All reported *P*-values were two-sided, and *P* < 0.05 was considered to be statistically significant. Discrimination was measured by Harrell’s concordance index (C-index), which quantifies the likelihood of two random patients. The patient who relapsed for the first time had a higher possibility of interest event. The C-index was calculated by R version 4.0.2 via the survival and design packages. All statistical analyses were carried out using SPSS version 25 and R version 4.0.2.

## Results

### Patient characteristics

In the training cohort, 229 patients (153 male, 76 females; median age, 40 years [range 18–79]) met the inclusion criteria. Clinical features for all patients are summarized in Table 1. Fifty-seven patients (24.9%) presented with B symptoms. Most of the patients (132 cases, 57.6%) had advanced disease (Ann Arbor stages III-IV). A total of 107 patients had more than one extra-nodal involvement. According to the IPI score, a majority of the patients (137 cases, 59.8%) were scored 2–5, and 92 patients (40.2%) were scored 0–1. One hundred fifty-three patients (66.8%) were classified as high-risk group and 76 patients (33.2%) was stratified as low-risk group. A total of 152 patients (66.4%) received R-CODOX-M (rituximab, cyclophosphamide, vincristine, doxorubicin, high-dose methotrexate) based first-line chemotherapy, and 25 patients (10.9%) had received R-EPOCH (rituximab, etoposide, prednisolone, doxorubicin, cyclophosphamide and vincristine) chemotherapy. Twenty-eight patients (12.2%) were treated with R-CHOP (rituximab, cyclophosphamide, vincristine, doxorubicin and prednisolone) and high dose methotrexate-based regimens, 17 patients (7.4%) received R-Hyper CVAD (rituximab, cyclophosphamide, doxorubicin, vincristine and dexamethasone) as first line chemotherapy, and 7 patients (3.1%) were treated with miscellaneous anthracycline-based regimens. Twenty-nine patients (12.7%) and 9 patients (3.9%) had received radiation therapy and autologous hematopoietic stem cell transplantation (ASCT), respectively. Details are shown in Table 1.

**Table 1:** Baseline characteristics of patients

Characteristics	Training(***n*** = 229)	Validation(***n*** = 107)
**Age (years)**		
< 60	192(83.8%)	85(79.4%)
≥ 60	37(16.2%)	22(20.6%)
**Gender**		
Male	153(66.8%)	72(67.3%)
Female	76(33.2%)	35(32.7%)
**ECOG score**		
< 2	168(73.4%)	75(70.1%)
≥ 2	61(26.6%)	32(29.9%)
**Ann Arbor stages**		
I–II	97(42.4%)	50(46.7%)
III–IV	132(57.6%)	57(53.3%)
**B symptoms**	57(24.9%)	22(20.6%)
**Risk stratification**		
Low	76(33.2%)	41(38.3%)
High	153(66.8%)	66(61.7%)
**Bone marrow involved**	36(15.7%)	16(15%)
**Central nervous system (CNS) involved**	14(6.1%)	5(4.7%)
**Lymph-node involvement**	111(48.5%)	49(45.8%)
**Extranodal involvement sites**		
< 2	122(53.3%)	63(58.9%)
≥ 2	107(46.7%)	44(41.1%)
**EBV encoded small RNAs** (**EBERs**)(**+)**	48(21%)	28(26.2%)
**ALB** < 40 mg/L	92(40.2%)	58(54.2%)
**CRP** ≥ 10 mg/L	101(43.7%)	47(43.9%)
**LDH** ≥ 334 U/L	87(38%)	43(40.2%)
**d-NLR** ≥ 1.6	58(25.3%)	34(31.8%)
**PLT** < 254 × 10^9^/L	127(55.5%)	54(50.5%)
**International Prognostic Indexs (IPI) score** ≥ 2	137(59.8%)	61(57%)
**Radiation therapy**	29(12.7%)	12(11.2%)
**ASCT**	9(3.9%)	3(2.8%)
**CNS prophylaxis**	184(80.3%)	89(83.2%)
**Abdominal lesions**	137(59.8%)	58(54.2%)
**Chemotherapy regimen**		
R-CODOX-M	152(66.4%)	69(64.5%)
R-EPOCH	25(10.9%)	8(7.5%)
R-CHOP	28(12.2%)	15(14%)
R-Hyper-CVAD	17(7.4%)	12(11.2%)
Others	7(3.1%)	3(2.8%)

### Determination of cut-off values for serum markers

The ROC was used to identify optimal cut-off values for indexes. The area under the curve is presented in Table 2. The AUC for LDH and PLT was 0.708 and 0.654, respectively. The optimal cut-off values of CRP and albumin levels were 10 mg/L and 40 mg/L, respectively. By analyzing the specificity and sensitivity of each value, the optimal cutoff values of d-NLR, PLT and LDH were taken as 1.63, 254 × 10^9^/L and 334 U/L, respectively. Thus, patients were dichotomized into 2 categories: less than, and greater than or equal to the cut-off values.

**Table 2:** The AUC values of the variable were calculated for OS. Optimal cutoff values for each inflammatory biomarker

Marker	AUC	*P*	Optimal cutoff value
LDH	0.708	< 0.001	334
d-NLR	0.622	0.013	1.6
PLT	0.654	0.002	254
NLR	0.59	0.066	/
LMR	0.596	0.052	

### Univariate analysis and multivariate analysis

**Fig. 1:**
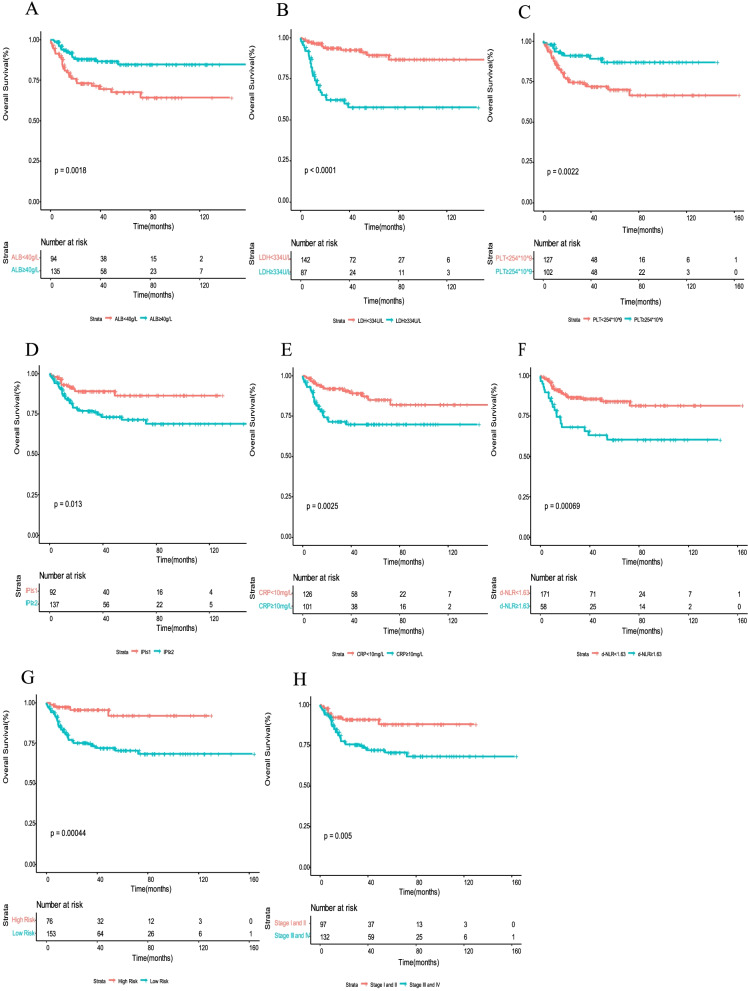
Kaplan-Meier survival analysis for OS according to optimal cut-off values of ALB (**A**), LDH (**B**), PLT (**C**), IPI score (**D**), CRP (**E**), d-NLR (**F**), risk stratification (**G**) and Ann Arbor stage (**H**)

Table 3 shows the results of univariate analysis of clinical variables considered predictors of OS. The following clinical factors significantly predicted poor survival in univariate analysis: ECOG≥2, bone marrow involvement, more than two extranodal involvement sites, advanced Ann Arbor stage (III/IV), IPI score ≥ 2, high risk stratification, with CRP ≥ 10 mg/L, ALB< 40 g/L, LDH ≥ 334 U/L, PLT < 254 × 10^9^/L, and d-NLR < 1.6. The univariate survival analysis for OS according to optimal cut-off values of ALB, LDH, PLT, d-NLR, CRP, IPI score, Ann Arbor stage, and risk stratification is shown in Fig. 1. Multivariate analysis was performed on clinical parameters related to shorter OS. We found that three variables maintained a negative prognostic influence on OS by using forward conditional Cox regression: ALB< 40 g/L (*P* = 0.041, hazard ratio (HR), 2.251; 95% CI, 1.034–4.902), LDH ≥ 334 U/L (*P* < 0.001, HR, 0.199; 95% CI, 0.081–0.488), and PLT < 254 × 10^9^/L (*P* = 0.038, HR, 2.261; 95% CI, 1.047–4.886) (Table 3).

**Table 3:** Univariate and multivariate analyses of potential prognostic factors for OS

	Univariate analysis		Multivariate analysis	
Characteristics	HR (95% CI)	P	HR (95% CI)	P
**Age**				
< 60				
≥ 60	0.754(0.35–1.626)	0.472	1.012(0.407–2.519)	0.979
**Gender**				
Male				
Female	0.502(0.24–1.046)	0.066		
**ECOG score**				
< 2				
≥ 2	4.457(1.973–10.069)	< 0.001	0.323(0.101–1.031)	0.056
**Ann Arbor stages**				
I–II				
III–IV	2.744 (1.316–5.723)	0.007	1.287(0.362–4.572)	0.696
**B symptoms**	1.155(0.593–2.252)	0.672	1.409(0.577–3.438)	0.452
**Risk stratification**				
Low				
High	5.214(1.863–14.594)	0.002	0.113(0.024–0.519)	0.005
**Bone marrow involved**	2.256 (1.158–4.395)	0.017	2.136(0.799–5.71)	0.13
**CNS involved**	0.469(0.184–1.194)	0.112		
**Lymph-node involvement**	1.081(0.594–1.966)	0.799		
**Extranodal involvement sites**				
< 2				
≥ 2	2.143(1.144–4.014)	0.017	0.841(0.351–2.016)	0.698
**EBERs**	0.147(0.035–0.61)	0.008	9.825(1.31–73.703)	0.026
**ALB <** 40 mg/L	0.387(0.208–0.718)	0.003	2.251(1.034–4.902)	0.041
**CRP** ≥ 10 mg/L	2.612(1.369–4.982)	0.004	1.124(0.494–2.557)	0.781
**LDH** ≥ 334 U/L	5.361(2.747–10.462)	< 0.001	0.199(0.081–0.488)	< 0.001
**d-NLR** ≥ 1.6	2.71(1.487–4.939)	0.001	0.501(0.246–1.019)	0.056
**PLT** < 254 × 10^9^/L	0.348(0.171–0.706)	0.003	2.261(1.047–4.886)	0.038
**IPI score** ≥ 2	2.376(1.171–4.822)	0.017	4.195(1.34–13.139)	0.014
**Radiation therapy**	0.928(0.391–2.203)	0.866		
**ASCT**	0.393(0.054–2.857)	0.356		
**CNS prophylaxis**	0.619(0.261–1.467)	0.276		
**Abdominal lesions**	1.152(0.612–2.166)	0.661

### Prognostic model construction and survival analysis

**Fig. 2:**
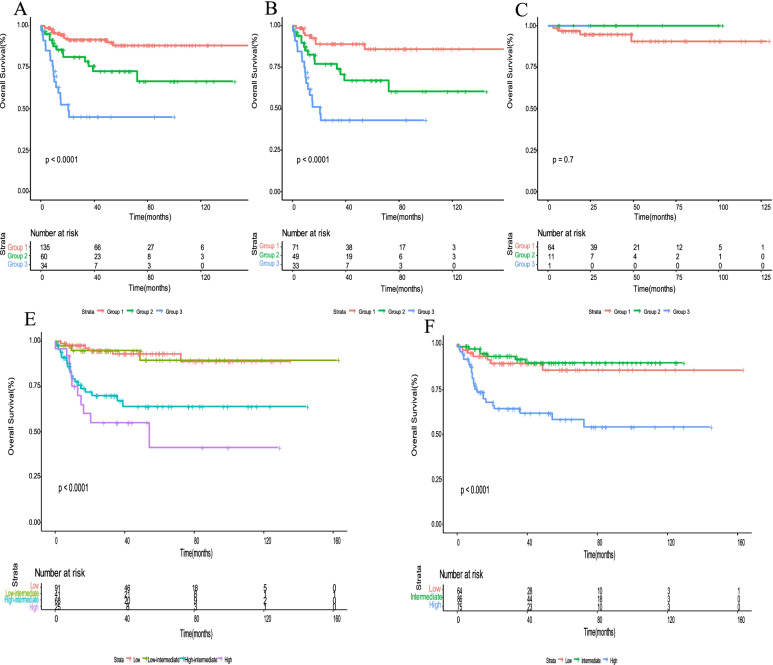
**A** Kaplan-Meier survival analysis for OS in Group 1,2 and 3 according to the new predictive model in the training cohort. **B** Kaplan-Meier survival analysis for OS in Group 1,2 and 3 for patients at high-risk in the training cohort. **C** Kaplan-Meier survival analysis for OS in Group 1,2 and 3 for patients at low-risk in the training cohort. **D** OS according to IPI score are shown. (low risk:0–1; low-intermediate risk:2; high-intermediate risk:3; high risk:4–5). **E** OS according to BL-IPI score are shown (low risk:0; intermediate risk:1; high risk:2–4)

Consequently, based on these 3 independent prediction factors (ALB< 40 g/L, PLT < 254 × 10^9^/L, and LDH ≥ 334 U/L) for OS in the multivariate analysis, a new prognostic model for all 229 patients was constructed by combining factors as follows: Group 1 (135 cases, 59%), no more than one adverse factor; Group 2 (60 cases, 26.2%) two adverse factors; and Group 3 (34 cases, 14.8%), three adverse factors. (Fig. 2A). The new predictive model for BL effectively stratified patients by prognosis. The median OS of Group 1 and Group 2 was not reached, while the median OS in Group 3 was 17 months. The 5-year survival rates of Group 1, 2 and 3 were 88.1, 72.4, and 45%, respectively (*P* < 0.0001). Based on the risk stratification by Mead et al. [16], the subgroup analysis for high-risk and low-risk patients was shown in Fig. 2B and C. Our prognostic model results showed that the high-risk patients with no more than one adverse factor presented a 5-year survival rate of 85.9%, but patients with three adverse factors in high-risk group revealed a 5-year survival rate of 43.0% (*P* < 0.0001). No significant difference was shown in low-risk patients (Fig. 2C). The C-index is 0.768 (95%CI 0.705–0.830). According to the IPI score, low-and low-intermediate risk patients could not be distinguished (*P* = 0.8889) (Fig. 2D). Low-risk and intermediate-risk patients were also not distinguished based on BL-IPI score (*P* = 0.5045) (Fig. 2E).

### External validation and survival prediction

**Fig. 3:**
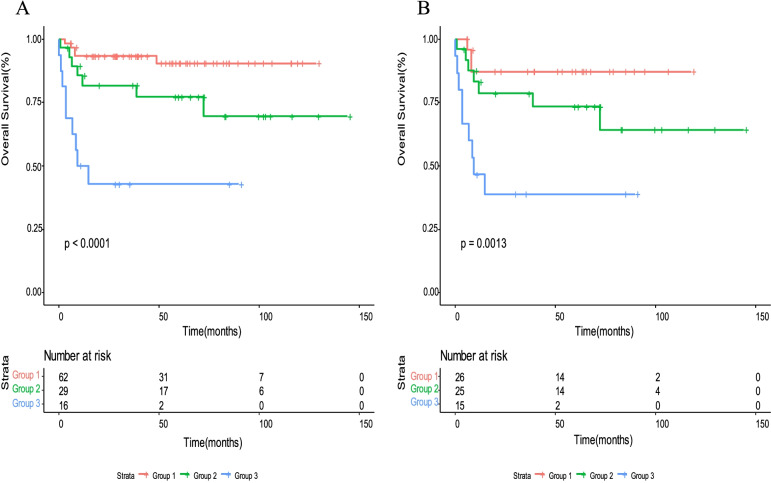
**A** Kaplan-Meier survival analysis for OS in Group 1,2 and 3 according to the new predictive model in the validation cohort. **B** Kaplan-Meier survival analysis for OS in Group 1,2 and 3 for patients at high-risk in the validation cohort

To validate our novel prognostic model, 107 patients from another four cancer centers were included. The characteristics of these patients are shown in Table 1. There was good consistency between the validation cohort and the training cohort. Three risk groups could also be predicted using the nomogram, and the 5-year survival rates of Groups 1, 2 and 3 were 90.5, 77.2, and 42.9%, respectively (*P* < 0.0001) (Fig. 3A). In the subgroup analysis for high-risk patients (Fig. 3B), our prognostic model showed that the high-risk patients with no more than one adverse factor presented a 5-year survival rate of 87.1%, but patients with three adverse factors in the high-risk group revealed a 5-year survival rate of 38.9% (*P* = 0.0013). The C-index is 0.806 (95%CI 0.727–0.887).

## Discussion

In our study, we summarized the clinical characteristics and pretreatment inflammatory biomarkers in 336 adult patients with sporadic BL. It was demonstrated that platelets, albumin and LDH independently predicted survival in univariate and multivariate analyses. Based on the value of platelets, albumin and LDH, a novel prognosis model was constructed and demonstrated a strong ability to stratify risks. External validation was performed with this newly-developed prognostic model. To our knowledge, we are the first to report prognostic model of adult sporadic BL based on real-world multicenter study data in the Asian population.

When the immune system is activated, immune-related cells secrete proinflammatory cytokines, leading to systemic inflammation [17]. Systemic inflammation has been shown to be an etiologic factor of advanced cancer [18]. Recent studies have found that cancer-related inflammation plays a key role in the progression of different malignancies. Inflammatory biomarkers, such as white blood cell counts and acute phase proteins, have repeatedly been shown to have prognostic value [19]. Prognostic markers of systemic inflammation, such as d-NLR, NLR and LMR, are associated with disease outcomes in a variety of tumors, and are readily available and inexpensive [20–27]. In our study, the variables of prognostic value were ALB, PLT and LDH, as independent indicators for sporadic BL. Our study revealed that in high-risk patients, the novel prognostic model could distinguish patients with significantly different survival rates. Patients at high-risk with no more than one adverse factor had a 5-year survival rate of 85.9%, which is similar to patients at low risk (5-year survival rate of 92.0%). Thus, we suggest that patients with no more than one adverse factor might receive de-escalation chemotherapy to avoid severe side effects.

Several studies have found negative correlations between elevated LDH and survival in patients with BL [28, 29]. The level of serum LDH in cancer patients has been reported to be an indicator of the cell proliferation rate and invasive potential [30]. In addition, LDH has been reported as a risk factor for tumor lysis syndrome (TLS) [31, 32]. However, the proper cut-off value for LDH to predict outcome was not clear. In the risk stratification for BL [33], patients would be at high risk if LDH was above the upper normal limit. In Andrew M’s study [34], LDH higher than 3 times the upper limit was associated with poor survival. Our study showed that LDH ≥ 334 U/L predicted adverse outcomes.

Serum albumin levels, as an excellent indicator of malnutrition and cachexia, have been widely used in various advanced cancers [35]. Proinflammatory cytokines such as interleukin (IL)-6, IL-1, and tumor necrosis factor, which regulate the production of hepatocyte albumin, causing a lower serum albumin concentration [36, 37]. Platelets have the ability to cause cell adherence in infections, inflammation and many malignant tumors [38]. Furthermore, they also mediate tumor cell growth, proliferation, and angiogenesis. Activated platelets can interact with cancer cells through paracrine signaling or direct contact to promote tumor cell growth and survival [39, 40]. In a recent study by Rachidi S et al., high platelet counts were associated with poor prognosis in patients with head and neck cancer, while patients with mid-normal platelet counts (230–314 × 10^9^/L) had a higher survival rate [41]. Suk-young Lee et al. analyzed 29 patients with intrahepatic cholangiocarcinoma who received gemcitabine plus cisplatin and found that platelet count≤180 × 10^9^/L was an independent prognostic factor for decreased OS [42]. In the current study, we found that platelet< 254 × 10^9^/L predicted adverse outcomes. This suggests that lower platelet level might be a marker of poor prognosis associated with BL. Further research will focus on the relationship between platelet counts and different cancer survival rates.

Peripheral LMR predicts survival outcomes in some hematologic malignancies and solid tumors such as diffuse large B-cell lymphoma, bladder cancer, soft tissue sarcomas and gastric cancer [43–46]. Stotz and colleagues reported that a low LMR predicted poor clinical outcome in stage III colon cancer patients [47]. Porrata et al. found that patients with high peripheral blood LMR before and during treatment of classical Hodgkin’s lymphoma had better OS [48]. Wang et al. showed that low LMR predicted poor outcome in 62 sporadic BL patients [15]. In our study, with a larger sample size, LMR was not associated with BL. Further study is need to confirm the underlying mechanism between LMR and BL. Proctor et al. reported that the d-NLR, which is based on leukocyte and neutrophil counts, had a prognostic value similar to that of the NLR. It could be recommended for risk stratification in chemotherapy patients [49]. The prognostic value of the d-NLR was further confirmed in subsequent studies in various types of cancers [50–52]. In our investigation, it was not associated with patient outcomes in multivariate analysis. We will expand the sample size to test our results in future studies.

In general, the IPI was shown to be a reliable prognostic indicator in aggressive lymphomas, taking into account the Ann Arbor stage, B symptoms, ECOG score, LDH and the extent of extranodal involvement, which were without significant prognostic value in our study. Similarly, the BL-IPI did not have a good risk stratification in our study. Because sporadic BL is considerably different from other aggressive lymphomas clinically and biologically, the IPI and BL-IPI might not be ideal for predicting OS in sporadic BL. Risk stratification has been widely used in BL patients, but the exploration of risk factors remains important. Mussolin et al. analyzed the long-distance polymerase chain reaction product in children with BL and developed a poor-prognosis subgroup among patients with high-risk BL [33]. In our study, we constructed a novel prognostic model for adult sporadic BL patients, including ALB, PLT and LDH, which was able to distinguish three groups with strikingly different survival rates.

Our study had some limitations of note. One limitation of this study lies in its retrospective nature and its heterogeneity in baseline risk and treatment factors, which may have led to potential bias. Furthermore, in the multivariate analysis, the prognostic values of tumor grade, CNS invasion and bone marrow invasion were not statistically significant, possibly due to the limited number of patients. Finally, these preliminary results needed to be further validated in larger prospective studies to clarify the mechanisms.

In summary, this study identifies pretreatment serum levels of ALB, PLT and LDH as clinically useful markers, which are inexpensive and readily available, to stratify patients into different risk groups that call for different treatments. Future studies involving larger patient groups with longer follow-up periods are needed to verify the results.

## Supplementary Information

**Additional file 1: Supplementary Table 1.** The AUC values of the variable were calculated for PFS.

## Data Availability

The datasets generated during the current study are available from the corresponding author on reasonable request.
