# Supplementary material for: A novel prognostic index for sporadic Burkitt lymphoma in adult patients: a real-word multicenter study
Source: BMC Cancer. 2022 Jan 7;22:45. doi: 10.1186/s12885-021-09144-1 (PMC8740497; doi:10.1186/s12885-021-09144-1)
Supplement: Supplementary file 1 — Additional file 1: Supplementary Table 1. The AUC values of the variable were calculated for PFS. [file 12885_2021_9144_MOESM1_ESM.docx]

**Supplementary table1 The AUC values of the variable were calculated for PFS.**

| Marker | AUC | *P* | Optimal cutoff value |
| --- | --- | --- | --- |
| LDH | 0.681 | <0.001 | 324.9 |
| d-NLR | 0.599 | 0.017 | 1.56 |
| PLT  NLR  LMR | 0.592  0.561  0.594 | 0.025  0.14  0.024 | 157.5  3.46 |
